# Welding fume inhalation exposure and high-fat diet change lipid homeostasis in rat liver

**DOI:** 10.1016/j.toxrep.2020.10.008

**Published:** 2020-10-11

**Authors:** Greg R. Boyce, Mohammad Shoeb, Vamsi Kodali, Terence G. Meighan, Katherine A. Roach, Walter McKinney, Samuel Stone, Matthew J. Powell, Jenny R. Roberts, Patti C. Zeidler-Erdely, Aaron Erdely, James M. Antonini

**Affiliations:** a National Institute for Occupational Safety and Health, Morgantown, WV, USA; b New River Laboratories, Morgantown, WV, USA

**Keywords:** Lipids, Diet, Welding fumes, Mass spectrometry

## Abstract

It is estimated that greater than 1 million workers are exposed to welding fume (WF) by inhalation daily. The potentially toxic metals found in WF are known to cause multiple adverse pulmonary and systemic effects, including cardiovascular disease, and these metals have also been shown to translocate to the liver. This occupational exposure combined with a high fat (HF) Western diet, which has been shown to cause hyperlipidemia and non-alcoholic fatty liver disease (NAFLD), has the potential to cause significant mixed exposure metabolic changes in the liver. The goal of this study was to use matrix assisted laser desorption ionization imaging mass spectrometry (MALDI-IMS) to analyze the spatial distribution and abundance changes of lipid species in Sprague Dawley rat liver maintained on a HF diet combined with WF inhalation. The results of the MALDI-IMS analysis revealed unique hepatic lipid profiles for each treatment group. The HF diet group had significantly increased abundance of triglycerides and phosphatidylinositol lipids, as well as decreased lysophosphatidic lipids and cardiolipin. Ceramide-1-phosphate was found at higher abundance in the regular (REG) diet WF-exposed group which has been shown to regulate the eicosanoid pathway involved in pro-inflammatory response. The results of this study showed that the combined effects of WF inhalation and a HF diet significantly altered the hepatic lipidome. Additionally, pulmonary exposure to WF alone increased lipid markers of inflammation.

## 1 Introduction

Inhalation of welding fumes (WF) is a common occupational exposure. It has been estimated that 1.2 million workers are exposed to WF daily in the U.S. [1,2]. Even greater numbers of welders, believed to be in the millions, are exposed to WF worldwide. Generated WF are a mixture of micro- and nanoparticles composed of different potentially toxic metals, such as iron, manganese, chromium, and nickel [[3], [4], [5]]. The composition of the resulting welding fume is mostly dependent on the consumption of the welding electrode [6]. The toxicity of inhaled welding fumes has been shown to be mostly dependent on the type of welding electrode and specific welding parameters used [7,8]. Exposure to WF are known to cause of a variety of pulmonary disorders, such as lung cancer, bronchitis, pulmonary function changes, and metal fume fever [1]. Welding fume exposure has also been shown to increase the incidence of cardiovascular diseases, including: acute myocardial infarct, angina pectoris, chronic ischemic heart disease, cardiac arrest, and heart failure, in exposed workers [9]. Less is known about the extra-pulmonary effects in other organ systems, specifically the liver. Metals associated with WF have been shown to translocate to liver. Chromium accumulated in the livers of Sprague-Dawley rats after a weekly exposure to WF [10]. Also, acute inhalation exposure to WF has been observed to cause oxidative damage in the liver of rats as evidenced by increases in both DNA strand breakage and lipid peroxidation [11]. Manganese and iron are also found in welding fumes and are important cofactors for catalyzing many metabolic processes, such as insulin signaling and lipid metabolism [12]. The translocation of these metals from the lungs to the liver, after inhalation exposure to a welder, may also alter the hepatic lipid metabolism and insulin resistance [13].

Obesity is also important factor in the acute severity of cardiovascular disorders after exposure to fine metal particulates found in welding fumes [14]. A modern, high fat (HF) western diet consists of elevated levels of fats, carbohydrates, protein, and sodium [15]. Long-term consumption of a HF, western diet can predispose individuals to various metabolic diseases, such as hyperlipidemia, diabetes, and cardiovascular disease [16]. The intake of a HF diet has been shown to cause an increase in lipid peroxidation in liver tissue and can also lead to non-alcoholic fatty liver disease (NAFLD) and hepatic steatosis [17]. The liver serves as the central organ for lipid homeostasis across many different organisms, and alterations in the species, concentrations, and oxidation of lipids within the liver have been shown to be indicators of hepatic injury or disease [18]. The increased storage of lipids in hepatic cells, known as steatosis, causes an increase in the occurrence of fibrosis, inflammation, cirrhosis, and hepatic cancer [19]. NAFLD and hepatic steatosis has also been shown to increase the prevalence of cardiovascular disease [20].

Previously, our group reported that the combination of an exposure to inhaled WF and maintenance on a HF western diet increased lipid accumulation in the livers of exposed rats as well as the serum concentrations of alanine transferase (ALT) and aspartate transferase (AST), enzymes used to assess liver changes [21]. To date, there is no information on the combined effects of an WF and HF diet maintenance on the hepatic lipidome, which is important because the obesity, metabolic disorders, and exposure to fine metal particulate are all factors in the severity of cardiovascular disorders that could directly impact exposed welders. The goal of the current study was to utilize matrix assisted laser desorption ionization imaging mass spectrometry (MALDI-IMS) to examine the abundance and spatial distribution of lipids in livers from animals exposed by inhalation to WF and fed a HF western diet. The results of this study will provide valuable insight by which an environmental/occupational exposure and lifestyle choice (*e.g.*, diet) potentially change the hepatic lipidome.

## 2 Methods

### 2.1 Animals and diet

Male Sprague-Dawley rats were acquired from Hilltop Lab Animals (Scottdale, PA) at 5 wk of age and were free of viral pathogens, parasites, mycoplasmas, *Helicobacter*, and *CAR Bacillus*. Rats were fed *ad libitum* with water and irradiated Teklad 2918 regular (REG) rodent diet (Envigo Teklad Diets, Madison, WI) and acclimated for 1 wk in an animal facility that is specific pathogen-free, environmentally controlled, and accredited by AAALAC, International (Frederick, MD). Following the acclimation period, half of the rats were continued on the Teklad 2918 REG diet. A second set of rats was started *ad libitum* on a custom 45 % high fat (HF) Kcal, Western Diet (Envigo Teklad Diets, Madison WI). The Western Diet was supplemented with 21 % anhydrous milk fat and 34 % sucrose. Soybean (2 %) was added to the HF diet to provide essential amino acids. Nutritional composition of the HF diet was 14.8 % protein, 40.6 % carbohydrate, and 44.6 % fat. All animal procedures used during the study were reviewed and approved by the CDC/NIOSH-Morgantown Institutional Animal Care and Use Committee. All methods were performed in accordance with the relevant guidelines and AAALAC International.

### 2.2 Welding fume exposure

At wk 7 during maintenance on the HF and REG diets, rats from each diet were exposed by inhalation to stainless steel WF (target concentration of 20 mg/m^3^ × 3 h/d × 4 d/wk × 5 wk) or filtered air (control). Four experimental groups (n = 6 per group) were assessed in the study: (1) HF + WF, (2) HF + Air, (3) REG + WF, and (4) REG + Air. At wk 12, rats were humanely euthanized by an intraperitoneal injection of sodium pentobarbital euthanasia solution (>100 mg/kg body weight; Fatal-Plus Solution, Vortech Pharmaceutical, Inc., Dearborn, MI) followed by exsanguination. The WF aerosol generator and animal exposure system [7] as well as the characterization of the Stainless Steel (SS)fume [21] were previously described. Briefly, WF was composed (weight %) of Fe (57 %), Cr (20.2 %), Mn (13.8 %), Ni (8.8 %) and Cu (0.2 %). as determined by inductively coupled plasma atomic emission spectroscopy according to NIOSH method 7300 [22]. The mass median aerodynamic diameter was 0.26 μm with a geometric standard deviation of 1.4 as determined by a Micro-Orifice Uniform Deposit Impactor (MOUDI, MSP Model 110, MSP Corporation, Shoreview, MN) and a Nano-MOUDI (MSP Model 115). The actual animal chamber concentration (mean + standard deviation) for the exposures was 20.3 ± 6.4 mg/m^3^.

### 2.3 Liver tissue preparation

Extracted livers from each group were flash frozen in liquid nitrogen and stored at −80 °C until cryo-sectioning and MALDI analysis. Fresh frozen livers were sectioned at 10 μm thickness on a NX70 Cryostar cryostat (Thermo Scientific, San Jose, CA) with a maintained temperature of −20 °C. Liver sections were then thaw mounted on indium tin oxide (ITO) slides (Delta Technologies, Loveland, CA) and desiccated for 12 h. Samples for MALDI-IMS analysis were scanned on an Epson V19 flatbed scanner prior to matrix application for teaching locations prior to mass spectrometry analysis.

Serial sections of each liver were cut at 5 μm thickness and thaw mounted to glass slides for Oil-red-O staining. Slides were air dried at room temperature for 10 min prior to staining. Slides were stained with Oil-Red-O for 10 min at 60 °C in an oven and then rinsed in distilled water. Propylene glycol rinses were then used for differentiation and then nuclei were stained with Gill’s hematoxylin solution for 30 s.

### 2.4 Matrix application

Liver slides were sprayed with 2,5-dihydroxybenzoic acid (DHB) (Sigma Aldrich, Darmstadt, Germany). DHB matrix was dissolved in 70 % acetonitrile (Thermo Scientific, San Jose, CA) at a concentration of 40 mg/mL for negative ion mode analysis, positive ion mode analysis used identical matrix solvent with the addition of 10 mM KCl. A TM-Sprayer (HTX Technologies, North Carolina) was used for matrix application. The TM sprayer was operated with a 0.1 mL/min flow rate in the serpentine deposition pattern with a 10 s drying time. The matrix was applied in 6 passes at a temperature of 75 °C. In order to reduce variability between slides, all slides were sprayed together.

### 2.5 MALDI imaging mass spectrometry (IMS)

Liver sections from each group were analyzed using a RapiFlex MALDI-ToF/ToF mass spectrometer (Bruker, Bremen, Germany). The laser was operated at 1 khz with 500 shots collected per location and spectra averaged for each location. Imaging locations were defined using the polygonal region tool in flexAnalysis software ver. 3.0. The spatial resolution was set to 100 μm for each imaging analysis. Prior to analysis the mass spectrometer was calibrated, and laser energy was adjusted to optimize signal intensity for the selected mass range. Tissues were analyzed in both positive and negative ion modes. The mass range for the imaging experiments was set at 500−1500 Da.

### 2.6 MALDI data analysis

MALDI IMS data were processed using flex-Imaging software (Bruker, Bremen Germany). Mass images were generated for select lipid *m/z*’s. The data was normalized to root mean squares (RMS) for each image. Mass images were then exported as. tiff files for publication. Principal component analysis was performed on every spectrum collected from the imaging analysis and processed using SCiLs Pro (Bruker, Bremen, Germany) software package.

### 2.7 Statistical analysis and principal component analysis

Mean peak intensities for each tissue and every location were imported into SCiLS Lab 2019c Pro software (Bruker, Bremen, Germany) for statistical analysis. The spectral data generated for the control (RD + Air) and all other treatments were combined into a single data file for each analysis (n = 3) in the SciLS software. The spectral data was normalized to total ion count (TIC). Principal component analysis was performed on the normalized spectral data and a pareto scaling was applied to the data. A one-way analysis of variance (ANOVA) was performed on the *m/z* intensity values shown to be changed from the SciLS analysis to determine significance. Significance was determined by a p-value of <0.05.

## 3 Results

**Fig. 1:**
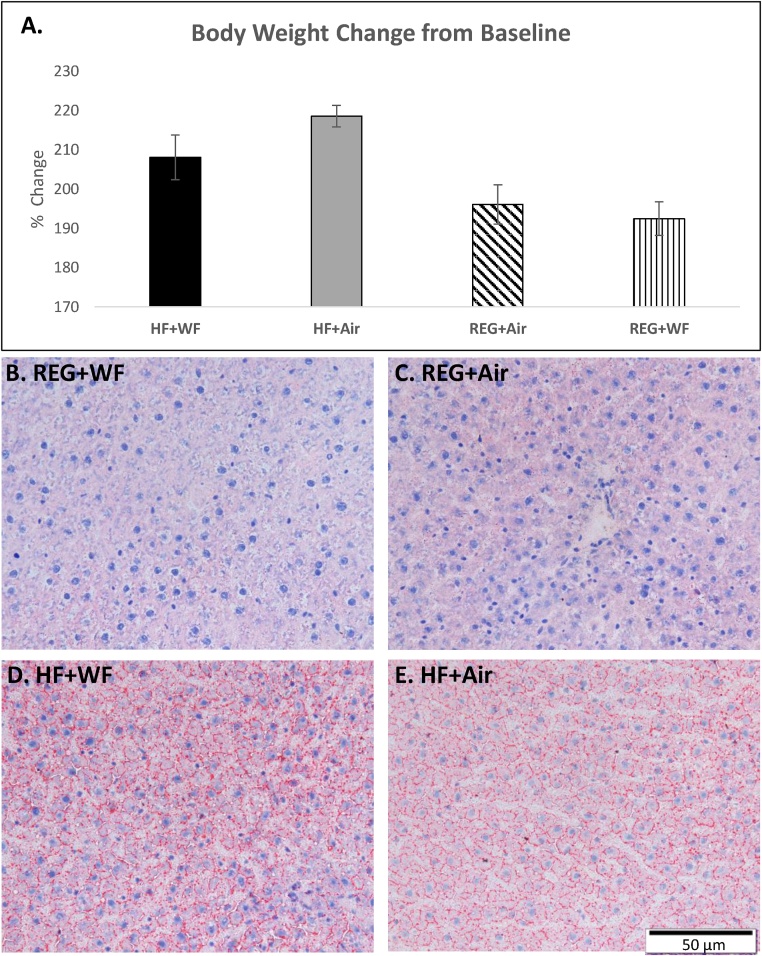
(A) Body weight percent change from baseline for all four treatment groups at 12 wk. Error bars represent standard error. (B–E)Snap frozen liver sections at 12 wk from REG + WF (B), REG + Air (C), HF + WF (D), and HF + Air (E) groups. Images taken at 40x magnification. Bar =50 μm.

**Fig. 2:**
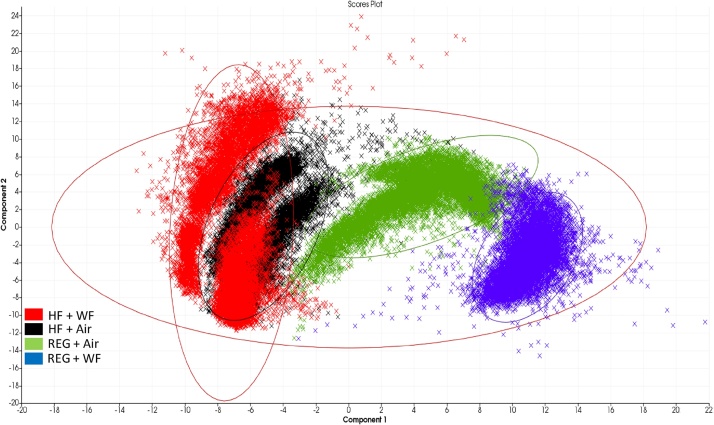
Principal component analysis of all spectra collected from spatial points across all liver tissues (n = 3 per group) at 12 wk for the HF + WF (red points), HF + Air (black points), REG + Air (green points), and REG + WF (blue points) groups. Ellipses represent the 95 % confidence interval.

**Fig. 3:**
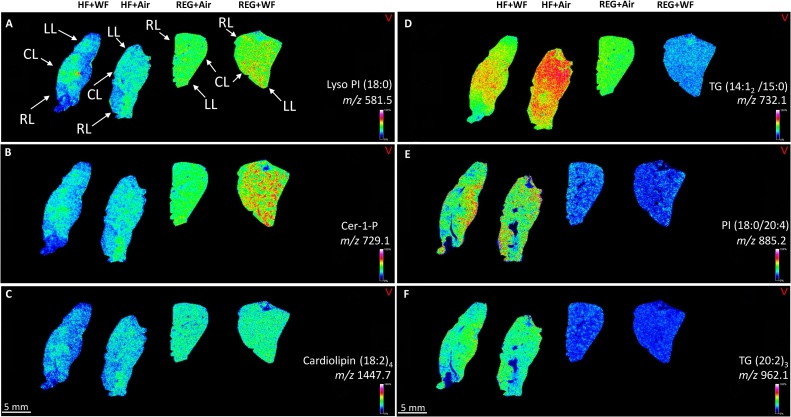
Ion maps showing spatial distribution and relative abundance of six lipids in livers at 12 wk from the 4 treatment groups. Lyso PI (18:0) *m/z 585.2* (A), Cer-1-P *m/z* 729.1 (B), Cardiolipin (18:2)_4_*m/z* 1447.7 (C), TG (14:1_2_ /15:0) *m/z* 732.1 (D), PI (18:0/20:4) *m/z* 885.2 (E), TG (20:2)_3_*m/z* 962.1 (F). LL = Left Lobe RL = Right Lobe CL = Caudate Lobe.

**Fig. 4:**
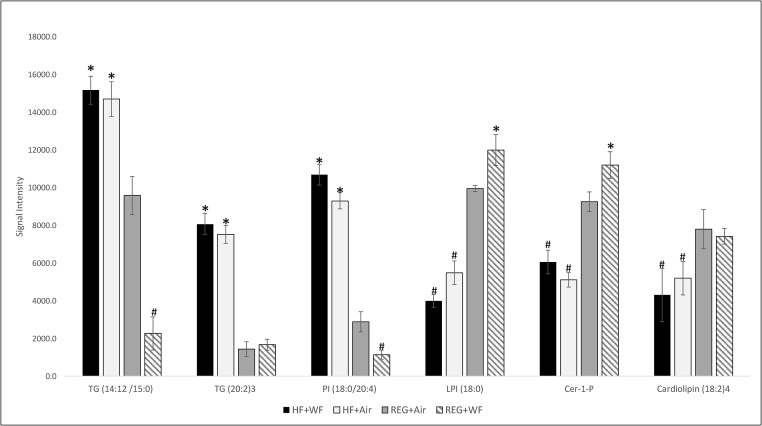
Liver lipids relative abundance based on MALDI signal intensity from rats exposed to welding fumes or air and fed either high fat (HF) or regular diet (REG) at 12 wk. Values are signal intensity means (n = 3), and standard error bars represent standard deviation. * indicate corresponding lipids within a group that are significantly higher (p < 0.05) than regular diet + air (REG + Air) and # indicate corresponding lipids within a group that are significantly lower (p < 0.05) than regular diet + air (REG + Air).

The serial liver sections stained with Oil-Red-O illustrated increased lipid deposition (red staining) in both HF groups (HF + WF and HF + Air) compared with the rats maintained on the REG diet (REG + WF and REG + Air) (Fig. 1A–D). The percent body weight change from baseline measurements showed the highest body weight increase was observed in the HF + Air group, while the lowest was found in the REG + WF group (Fig. 1E). The MALDI-IMS analysis yielded unique lipid profiles for each treatment group. These unique lipidome groups were visualized in the principal component analysis (PCA) score plots (Fig. 2). There were clear separations between the two REG diet groups. However, there was visible overlap in the lipid profiles generated from the HF + WF with a subset of the HF + Air group. The MALDI-IMS analysis revealed differing lipid distributions across all four treatment groups in the right lobe (RL), left lobe (LL), and caudate lobe(CL) of the livers (Fig. 3). The HF diet groups had lower abundance of shorter chain lyso-phosphatidylinositol (18:0) in their livers compared to the REG diet groups, whereas the abundance of this monoaryl lipid was higher in the REG + WF group than the REG + Air groups (Fig. 3A). Ceramide 1-phosphate (Cer1P) was observed at very high abundance in the liver of the REG + WF group when compared to the other three treatments (Fig. 3B). The HF diet groups had increased distribution of multiple triacyl glycerides. The two triacyl glycerides with the highest increase in signal across the liver tissue of the HF diet fed animals when compared to the REG + Air group were TG (14:1_2_ /15:0) and TG (20:2)_3_ (Fig. 3C/D). These two TGs were found to be in significantly higher abundance in the HF diet animals than in the REG diet animals (Fig. 4). However, TG (14:1_2_ /15:0) was shown to be in significantly lower abundance in the liver of the REG + WF group compared to the REG + Air group (Fig. 4). The phosphatidylinositol lipid PI (18:0/20:4) was in higher abundance in the liver of the HF diet groups compared to the REG diet groups (Fig. 4).

## 4 Discussion

In this study, we utilized MALDI-IMS to examine the abundance and spatial distributions of lipids in the livers of rats exposed to WF through inhalation while being maintained on a REG or HF western diet. The Oil-Red-O staining revealed the presence of steatosis in the livers of both the HF + Air and HF + WF groups, while the REG diet groups showed no signs of excess lipid deposition in the liver tissue regardless of air or WF exposure. This indicated that WF exposure alone did not substantially impact the accumulation of excess lipid in the liver. After 12 wk of diet maintenance and a 5-wk WF exposure, MALDI-IMS analysis revealed the hepatic lipidomes were unique based on either inhalation exposure or diet. The two groups fed the REG diet clustered separately as assessed by PCA with the REG + WF group standing alone; however, there was overlap observed between the two HF diet groups (HF + Air and HF + WF).

MALDI-IMS analysis of the liver tissues showed a significant increase in the two triglycerides, TG (14:1_2_ /15:0) and TG (20:2)_3_, in the HF diet groups. The increase in these triglycerides was observed ubiquitous across both HF diet groups, and this distribution aligned with the results of lipids in the oil-red-o stained liver sections. Abundance of these two triglycerides was not significantly different between the air- and WF-exposed HF diet animals; however, TG (14:1_2_ /15:0) was significantly lower in the REG + WF group when compared to the REG + Air control group. Triglycerides that are stored in the liver are primarily derived from food intake, and the reduced abundance of this triglyceride in the REG + WF group could be caused by decreased food intake, as seen by the slight decrease in body weight for that group [23]. The significant increase of triglycerides in the HF groups is not surprising, as it has been shown that these lipids accumulate in liver tissues of animals maintained on this type of diet [24]. Triglyceride metabolism in the liver occurs by hydrolysis of the triglyceride to diglyceride by the adipose triglyceride lipase (ATGL) enzyme, which then is followed by a hydrolysis of the resulting diglyceride into a monoglyceride by the hormone sensitive lipase (HSL) enzyme [23]. Duarte et al. showed that a HF diet suppressed de novo lipogenesis in mice, but it did not suppress triglyceride biosynthesis [25]. This alteration of the lipid biosynthesis pathways could explain the significant increase of triglycerides observed in the HF diet groups compared the REG diet groups. The observed dyslipidemia in HF Diet groups leads to NAFLD which has been shown to increase the risk of cardiovascular disease [20]. NAFLD has been shown to increase the severity and mortality of cardiovascular disease through increased abdominal weight, hypertension, and insulin resistance [26].

The HF diet groups showed significantly lower abundance of lyso-PI (18:0) than the REG diet groups. Lysophospholipids are monoglycerides that serve multiple functions including: cellular membrane structure, cell signaling, and lipid homeostasis [27]. The decrease in the abundance of lyso-PI (18:0) in the HF diet groups indicated a possible disruption in the hepatic lipid homeostasis, which was confirmed by the presence of steatosis shown by the Oil-Red-O staining. In comparing the REG + Air and REG + WF groups, the WF-exposed animals demonstrated a significantly higher abundance of lyso-PI (18:0) than the REG + Air group. It has been shown that increased levels of lyso-PI (18:0) in liver tissue is an indicator of hepatic inflammation [28]. The significantly higher abundance of this lipid in the livers of REG + WF exposed rats compared to all other treatments indicated the inhalation exposure to WF likely resulted in hepatic inflammation.

Ceramide-1-phosphate (Cer1P) was found in significantly higher abundance in the REG + WF group than the other treatment groups. Cer1P is a sphingolipid that is involved in multiple biological pathways. Interestingly, Cer1P plays a crucial role in signaling for the eicosanoid pathway [29]. The eicosanoid pathway is a primary mediator in the initiation and resolution of inflammation [30]. The increased level of Cer1P in the REG + WF group indicated the presence of hepatic inflammation and was supported by the increased level of serum AST observed in our previous study [21] Ceramide metabolism has also been shown to be disrupted by an excess of saturated fatty acids and this would explain the lower abundance of Cer1P in the HF diet groups [31].

Finally, the significant decrease in Cardiolipin (18:2)_4_ abundance in the HF diet animals compared to the REG diet groups regardless of exposure to air or WF may be explained through cardiolipin remodeling [32]. Sullivan et al. [32] showed that mice fed a HF diet displayed changes in the acyl chain lengths of their hepatic cardiolipins, and that acyl chain length remodeling is a process that occurs to maintain mitochondrial function in the presence of excess lipids.

To summarize, the results of this study showed that the combined effect of diet and inhalation exposure influenced the hepatic lipidome of rats. The most significant and pronounced changes were observed in the abundance of multiple triglycerides in the livers of the animals maintained on the HF western diet. However, the increase of Cer1P in the REG + WF group showed the upregulation of the pro-inflammatory eicosanoid pathway. The influence of WF inhalation on the hepatic lipidome was most pronounced when not combined with the lifestyle influence of the HF diet. There are definitive interactions between the HF and WF effects, but the responses are less prominent in this group showing that the diet effect has the greater influence on the lipidome. This study revealed that occupational exposures, with and without HF diet, can influence the hepatic lipidome and lipid metabolism. Further investigation is warranted into the influence on primary and /or secondary health effects of the combined occupational and lifestyle exposure.

## Author statement

All authors contributed equally in the preparation of the manuscript.

## Disclaimer

The findings and conclusions in this report are those of the authors and do not necessarily represent the official position of the National Institute for Occupational Safety and Health, Centers for Disease Control and Prevention.

## Declaration of Competing Interest

The authors report no declarations of interest.
